# Few Pharmacologic Interventions Prevent or Mitigate Frailty: A Systematic Review

**DOI:** 10.1093/geroni/igaf122.3592

**Published:** 2025-12-31

**Authors:** Leah Bolles, Michael DeGloria, Hailey Cray, Rebekah Harris, Saadia Qazi, Ariela Orkaby

**Affiliations:** VA Boston Healthcare System, Boston, Massachusetts, United States; VA Boston Healthcare System, Boston, Massachusetts, United States; VA Boston Healthcare System, Boston, Massachusetts, United States; VA Boston Healthcare System, Boston, Massachusetts, United States; VA Boston Healthcare System, Boston, Massachusetts, United States; Brigham & Women’s Hospital, Harvard Medical School, Boston, Massachusetts, United States

## Abstract

Frailty is a multidimensional clinical syndrome which limits the ability of older adults to cope with both everyday and acute stressors. To date, most interventions for frailty target lifestyle improvements, however, uncertainty remains as to whether pharmacologic interventions may prevent or mitigate this syndrome. Therefore, this systematic review aims to explore the efficacy of existing pharmacologic options for the treatment of frailty. Following PRISMA guidelines, we conducted our search using PubMed, CINAHL, and ClinicalTrials.gov from article inception to 10 June 2025. Only randomized controlled trials evaluating the effect of pharmacologic interventions on validated frailty measures were included. Two authors independently screened articles and extracted data, and disagreements were resolved through a third author. After screening 747 titles and abstracts, 33 full text manuscripts were extracted, and 10 trials were included. Results indicated varying efficacy across unique pharmacologic interventions. L-carnitine, protein supplementation at higher doses, testosterone therapy, and intravenous allogenic mesenchymal stem cells show promise in preventing and/or reducing frailty. Prebiotic administration, aspirin, vitamin D3 with omega-3 fatty acids, canakinumab, and metformin showed no reduction in overall or incident frailty. Notably, the combined supplementation of vitamin D3, omega-3s, and a structured exercise program reduced the risk of becoming pre-frail. Data were insufficient to conduct a meta-analysis. Although some pharmacologic interventions exhibit potential benefits, data remains limited in guiding therapeutic approaches to prevent or mitigate frailty in older adults.

